# Associations of Cholesin and GPR146 with atherosclerotic plaque stability: integrated bioinformatics and functional analyses

**DOI:** 10.3389/fcvm.2026.1808833

**Published:** 2026-08-07

**Authors:** Qing Liu, Yu Geng, Yifei Wang, Tingting Lv, Ping Zhang

**Affiliations:** 1 Clinical Medical College, Qinghai University, Xining, China; 2 Department of General Practice, Suining Central Hospital, Suining, China; 3 Department of Cardiology, Beijing Tsinghua Changgung Hospital, School of Clinical Medicine, Tsinghua University, Beijing, China

**Keywords:** atherosclerosis, bioinformatics analysis, Cholesin, GPR146, THP-1

## Abstract

**Background:**

Atherosclerotic plaque instability is a major determinant of acute cardiovascular events; however, reliable blood-based indicators reflecting plaque stability remain limited. Cholesin, a recently identified endogenous ligand of G protein–coupled receptor 146 (GPR146), has been implicated in cholesterol metabolism, but its potential relevance to human atherosclerosis and plaque stability remains largely unknown.

**Methods:**

We used real-time quantitative PCR (RT-qPCR) to measure Cholesin expression in peripheral blood mononuclear cells (PBMCs) from 60 participants, including patients with stable plaques, patients with unstable plaques, and controls. Public transcriptomic datasets (GSE100927, GSE28829, and GSE163154) were analyzed to characterize the expression patterns of Cholesin and GPR146 across plaque development and instability. Differential expression, co-expression, and functional enrichment analyses were performed. *In vitro*, THP-1 monocytes were stimulated with oxidized low-density lipoprotein (oxLDL), and the effects of GPR146 silencing on inflammatory responses were evaluated.

**Results:**

Cholesin mRNA expression in PBMCs was higher in patients with stable plaques than in those with unstable plaques. In contrast, Cholesin expression in plaque tissue was downregulated in atherosclerotic lesions compared with controls but did not differ significantly between disease stages or stable and unstable phenotypes. In the GSE28829 dataset, GPR146 expression was upregulated in advanced lesions, and in GSE163154, GPR146 expression was higher in unstable than in stable plaques. In THP-1 cells, oxLDL treatment increased GPR146 expression, whereas GPR146 silencing attenuated oxLDL-induced pro-inflammatory gene expression.

**Conclusion:**

PBMC Cholesin mRNA was associated with plaque phenotype, whereas plaque-tissue Cholesin did not distinguish lesion stage or stability. In contrast, GPR146 expression was higher in advanced and vulnerable plaques, and GPR146 silencing attenuated oxLDL-induced pro-inflammatory gene expression in THP-1 monocytes. These findings identify GPR146 as a candidate molecule associated with plaque progression and instability, although its causal role requires further validation.

## Introduction

Atherosclerotic cardiovascular disease remains the leading cause of death and disability worldwide, imposing a substantial burden on global health systems (1, 2). Atherosclerosis, the primary pathological process underlying cardiovascular disorders, is widely distributed in the coronary arteries, aorta, carotid arteries, and cerebral arteries (3). Despite advances in risk factor management and reperfusion therapy, the majority of acute ischemic events are triggered by the rupture of unstable plaques. Therefore, reliable strategies to identify and manage high-risk plaques are urgently needed (4, 5). Current clinical indicators such as luminal stenosis cannot fully reflect plaque vulnerability, and no widely accepted circulating biomarker is available to accurately identify plaque instability in individual patients.

G protein-coupled receptor 146 (GPR146) has recently emerged as a promising molecular target. Large-scale human genetic studies by Rimbert et al. demonstrated that loss-of-function variants in GPR146 are associated with a favorable cardiometabolic profile, including reduced LDL-C and a trend towards lower coronary artery disease risk, supporting GPR146 as a compelling new target for lipid-lowering therapy (6). However, the endogenous ligand responsible for modulating GPR146 activity *in vivo* remained unidentified. This critical gap was recently addressed by Hu et al. (7), who identified Cholesin (C7orf50) as an endogenous ligand for GPR146. They demonstrated that the Cholesin-GPR146 axis regulates hepatic cholesterol synthesis via the PKA-ERK1/2 signaling pathway, providing a mechanistic basis for the genetic observations.

Despite this crucial advance in basic science, the clinical and pathological implications of the Cholesin-GPR146 axis in human atherosclerosis remain largely unknown. It remains unclear whether this pathway exerts local effects within the plaque microenvironment, independent of its systemic metabolic effects.

Peripheral blood mononuclear cells (PBMCs) are easily accessible and have been widely used for transcriptomic biomarker discovery in cardiovascular diseases (8). Although Cholesin is primarily secreted by enterocytes, we hypothesized that PBMC Cholesin mRNA levels might reflect systemic metabolic or inflammatory status associated with plaque phenotype, thus serving as a surrogate indicator in this exploratory study. To address this question, an integrated bioinformatic and clinical investigation was performed. Public transcriptomic datasets were analyzed to characterize the expression patterns of Cholesin and GPR146 across different stages of AS, from initiation to progression and rupture. Furthermore, Cholesin expression was measured in clinical samples to evaluate its correlation with plaque phenotypic status.

## Materials and methods

### Study design

This study was conducted in accordance with the principles of the Declaration of Helsinki and was approved by the Institutional Ethics Committee of Beijing Tsinghua Changgung Hospital (Approval No. 23578-4-01). Written informed consent was obtained from all participants. A total of 60 participants were enrolled, including 24 patients with stable plaques, 24 patients with unstable plaques, and 12 angiographic controls without significant coronary stenosis. 24 individuals were allocated to the stable plaque group, which was characterized by the absence of recent acute ischemic events and specific OCT findings including a thick fibrous cap, absence of intraplaque hemorrhage, and absence of a lipid-rich necrotic core. 24 individuals were assigned to the unstable plaque group, defined by recent acute ischemic events related to the culprit lesion and/or OCT features of plaque vulnerability, including a thin fibrous cap, a large lipid core, intraplaque hemorrhage, or surface ulceration. Twelve individuals with no angiographic evidence of coronary artery stenosis served as the control group. Key exclusion criteria were as follows: active infectious diseases, autoimmune disorders, malignant tumors, severe hepatic or renal dysfunction, and use of immunosuppressive therapy.

### Real-time quantitative PCR analysis

Total RNA was isolated from THP-1 cells and PBMCs in the peripheral blood of patients using the Total RNA Extraction Kit (R1200, Solarbio, Beijing, China) in accordance with the manufacturer's instructions. Extracted RNA was reverse-transcribed into complementary DNA (cDNA) using the PrimeScript RT Master Mix (RR036A, Takara, Japan). Real-time quantitative polymerase chain reaction (RT-qPCR) was performed with a SYBR Green reagent kit (CN830A, Takara, Japan) on the real-time fluorescence PCR system (Bio-Rad, USA). The PCR program was as follows: 95 °C for 30 s, followed by 40 cycles of 95 °C for 5 s, and 60 °C for 30 s. Relative gene expression levels were calculated using the 2^−ΔΔCT^ method with β-actin as the internal reference gene for normalization. The sequences of the specific primers used in this study are presented in Table 1.

**Table 1:** PCR primer sequences.

Gene name	Primer sequence (5′-3’)
hCholesin-forward	AGCTGAAAAAGGAACGGAAGAA
hCholesin-reverse	TGGAGAAGTGCTCATCGGGAA
β-actin-forward	TGACGTGGACATCCGCAAAG
β-actin-reverse	CTGGAAGGTGGACAGCGAGG
IL-1β-forward	TGTGCTGAATGTGGACTCA
IL-1β-reverse	ACAAAAGGGCTGGGGAT
MCP-1-forward	TCTCGCCTCCAGCATGAAAG
MCP-1-reverse	GGCATTGATTGCATCTGGCT
TNF-α-forward	AGCAAGGACAGCAGAGGA
TNF-α-reverse	GGGGAGAGAGGGTGGAG
GPR146-forward	TGAGCCTCGACCACTACATC
GPR146-reverse	GCTTCTGCGTTCTGCATCTTG

### Data collection

Three publicly available gene expression datasets were obtained from the Gene Expression Omnibus (GEO) database (https://www.ncbi.nlm.nih.gov/geo/): GSE100927, GSE28829, and GSE163154. The GSE100927 dataset contained 35 control vascular samples and 69 atherosclerotic samples. GSE28829 included 13 early atherosclerotic plaque samples and 16 advanced atherosclerotic plaque samples. GSE163154 dataset comprised 16 non-intraplaque haemorrhage (non-IPH) samples and 27 intraplaque haemorrhage (IPH) samples.

### Differentially expressed gene screening

Differentially expressed genes (DEGs) were identified using the criteria of adjusted *P*-value <0.05 and |log2 (fold change)| > 1 for all datasets. DEG analysis was performed using the limma package, either via the GEO2R online tool (https://www.ncbi.nlm.nih.gov/geo/geo2r/) or locally in R when covariate adjustment was necessary. Visualization was performed using an online bioinformatics platform (https://www.bioinformatics.com.cn/).

### Co-expression analysis

To identify core genes significantly co-expressed with GPR146, Pearson correlation coefficients were calculated between GPR146 and all other genes using expression data from the GSE28829 and GSE163154 datasets. To validate robustness and account for potential confounding factors, two supplementary approaches were applied: (1) Pearson correlation analyses were performed using covariate-adjusted residual expression values derived from linear regression (gene–covariates); and (2) partial correlation analysis was conducted while controlling for the same set of confounding variables. All raw *P*-values were adjusted for multiple testing using the Benjamini–Hochberg false discovery rate (FDR) procedure within each dataset. The complete results, including correlation coefficients (*r*), raw *P*-values, and adjusted FDR values, are provided in Supplement 1. The top 10 positively and top 10 negatively correlated genes were selected for visualization. Correlation heatmaps were constructed using the ggplot2 package in R to illustrate expression relationships among these candidate genes.

### Functional enrichment analysis

To characterize the biological functions and signaling pathways associated with these co-expressed genes, gene ontology (GO) and Kyoto Encyclopedia of Genes and Genomes (KEGG) enrichment analyses were conducted using the DAVID database (9). GO enrichment was categorized into three functional modules: the biological processes (BP), cellular components (CC), and molecular functions (MF) (10), while KEGG pathway analysis was further implemented to identify the predominant signaling cascades enriched among the target genes (11). Enrichment significance was defined as a *P*-value <0.05. Visualization of enrichment results was generated using an online bioinformatics platform (https://www.bioinformatics.com.cn/).

### Cell culture and treatment

The human monocytic cell line THP-1 was obtained from the National Experimental Cell Resource Sharing Service Platform. Cells were cultured in RPMI 1640 medium (Corning, USA) supplemented with 10% fetal bovine serum (FBS) (Gibco, USA), and 1% penicillin–streptomycin solution, and maintained at 37 °C in a humidified atmosphere containing 5% CO_2_. Cells between passages 3 and 20 were used for all experiments.

For GPR146 silencing, THP-1 cells were transfected with GPR146-targeting siRNA or the corresponding negative-control siRNA. For GPR146 overexpression, cells were transfected with a pcDNA3.1-GPR146 expression plasmid or the corresponding empty pcDNA3.1 vector using LipoHigh transfection reagent (Sangon, China), according to the manufacturer's instructions. Twenty-four hours after transfection, cells were stimulated with 50 μg/mL oxidized low-density lipoprotein (oxLDL; Yiyuan, Guangzhou, China) for an additional 24 h. Cells were then harvested 48 h after transfection, and total RNA was extracted for subsequent analysis of inflammatory gene expression by RT-qPCR.

### GPR146 siRNA transfection

The small interfering RNAs (siRNAs) targeting GPR146 were designed and synthesized. The sequences were as follows: siGPR146-1, sense 5′-CGCUGGUGUUCAUCGGCUA-3′, antisense 5′-UAGCCGAUGAACACCAGCG-3′; siGPR146-2, sense 5′-CGGACGUGUACUUUGUCAA-3′, antisense 5′-UUGACAAAGUACACGUCCG-3′; siGPR146-3, sense 5′-GGAGCUGCAGCUGGUUCAA-3′, antisense 5′-UUGAACCAGCUGCAGCUCC-3′. A non-targeted siRNA (siNC) provided by the manufacturer was used as the negative control. Cells were seeded in antibiotic-free medium to reach approximately 70% confluence before transfection. Transfections were performed using a siRNA transfection reagent (Sangon, China). After 48 h, knockdown efficiency was verified by RT-qPCR and Western blotting, and the most effective siRNA was selected for subsequent experiments.

### Western blot

THP-1 cells under the different conditions were lysed by radioim-munoprecipitation assay (RIPA) buffer supplemented with protease and phosphatase inhibitors. Total protein concentrations were quantified using a BCA protein assay kit (absin, China). Equal amounts of denatured proteins were separated by 10% SDS-PAGE and transferred onto the polyvinylidene fluoride (PVDF) membranes (Cytiva Amersham™, Buckinghamshire, UK). Membranes were blocked with 5% non-fat dried milk in TBST buffer for 2 h on a shaker. Following blocking, membranes were incubated with primary antibodies overnight and then with secondary antibodies for 1 h, with continuous gentle shaking during both incubation steps. The immunoblots bands were visualized using ECL Plus western blotting substrate (Pierce, USA). Band intensities were quantified by ImageJ software. The following primary antibodies used were GPR146 (1:500, Sangon, China) and α-Tubulin (1:1000, Sangon, China).

### Statistical analysis

All statistical analyses were performed using the GraphPad prism 8.0 (GraphPad Prism Inc., Chicago, USA). Differences between two groups were analyzed using Student's *t*-test. For comparisons among three or more groups, one-way analysis of variance (ANOVA) followed by Tukey's *post-hoc* test was used. The Benjamini–Hochberg method was applied to correct for multiple testing where appropriate. Data are presented as mean ± standard deviation (SD). A *P*-value <0.05 was considered statistically significant.

## Results

### Cholesin mRNA expression in PBMCs from study participants

**Figure 1:**
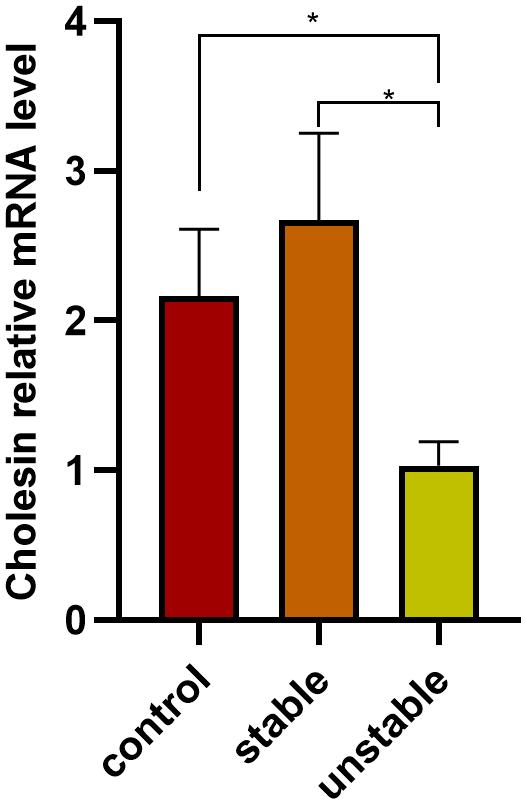
Relative mRNA expression levels of Cholesin in peripheral blood mononuclear cells (PBMCs) from study participants. RT-qPCR was performed to quantify Cholesin transcript levels among the control group (*n* = 12), stable atherosclerotic plaque group (*n* = 24), and unstable atherosclerotic plaque group (*n* = 24). Data are presented as mean ± standard deviation (SD). Statistical comparisons were conducted using one-way analysis of variance (*ANOVA*) followed by *Tukey's post-hoc test.* *P *<* 0.05 compared with the unstable plaque group.

To explore the clinical significance of Cholesin in atherosclerosis, we detected the mRNA expression levels of Cholesin in PBMCs from 60 participants including 24 patients in the plaque stable group, 24 in the unstable group, and 12 in the control group. RT-qPCR analysis revealed that Cholesin mRNA levels differed significantly across the three groups (*P* < 0.05). *Post-hoc* comparisons showed that Cholesin expression was significantly higher in the stable plaque group than in the unstable plaque group (*P* *<* 0.05) and significantly higher in the control group than in the unstable plaque group (*P* *<* 0.05). No significant difference was observed between the stable plaque group and the control group (Figure 1).

### The expression of Cholesin and GPR146 in plaque tissues

To assess heterogeneity across datasets, we summarized the basic information for GSE100927, GSE28829, and GSE163154, including sample size, age range, disease stage, tissue type, and transcriptomic platform (Table 2). GSE100927 comprises 104 samples from carotid, femoral, and infra-popliteal arteries, comparing atherosclerotic plaques vs. control vessels; GSE28829 includes 29 carotid plaque samples comparing advanced vs. early lesions; and GSE163154 includes 43 carotid endarterectomy samples comparing intraplaque hemorrhage (IPH) vs. non-IPH. Given the differences in sample origin, disease stage, and platform, we analyzed each dataset separately without cross-dataset merging.

**Table 2:** Summary of sample characteristics for the GSE100927, GSE28829, and GSE163154 datasets.

Variables	GSE100927	GSE28829	GSE163154
Disease stage	Atherosclerotic arteries vs. healthy arteries	Advanced lesions vs. early lesions	IPH vs. non-IPH
Sample size (*n*)	104	29	43
Age (years)	70.3 vs. 47.9	69.7 ± 8.1 vs. 69.9 ± 10.4	72.9 ± 6.3(all participants)
Tissue type	Carotid, femoral and infra-popliteal arteries	Atherosclerotic carotid artery segments	Carotid endarterectomy
Transcriptomic platform	GPL17077 (Agilent)	GPL570 (Affymetrix)	GPL6104 (Illumina)

IPH, intraplaque haemorrhage. Note for GSE163154: The original study (Jin et al., Circ Res 2021) reported the mean age for all 43 participants (23 males). Age data for IPH and non-IPH subgroups were not separately reported in the original study or the GEO record.

**Figure 2:**
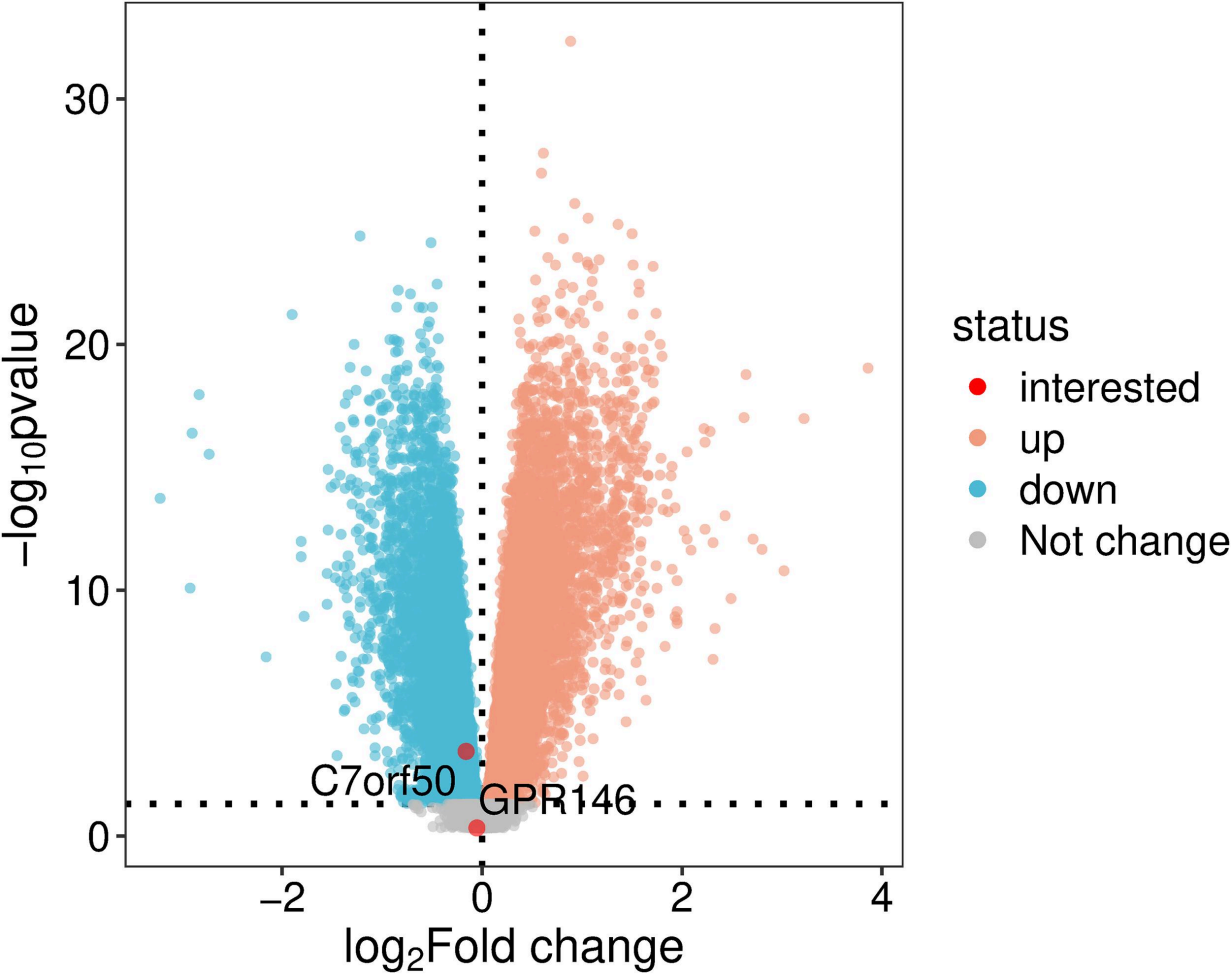
Volcano plot of differentially expressed genes in the GSE100927 dataset comparing atherosclerotic plaques with control vessels. Cholesin is highlighted in red and was significantly downregulated in atherosclerotic plaques compared with control vessels. Differentially expressed genes were defined using the following cutoff criteria: |log2FC| > 1 and adjusted *P* *<* 0.05 after Benjamini-Hochberg correction. The analysis included atherosclerotic plaque samples (*n* = 69) and control vessel samples (*n* = 35).

We first analyzed the GSE100927 dataset to compare gene expression profiles between atherosclerotic plaque tissue and normal control vascular tissue. Differential expression analysis showed that Cholesin expression was significantly downregulated in atherosclerotic plaque tissues relative to control vessels, whereas GPR146 showed no significant difference between the two groups (Figure 2).

### GPR146 is upregulated in advanced and vulnerable atherosclerotic plaques

**Figure 3:**
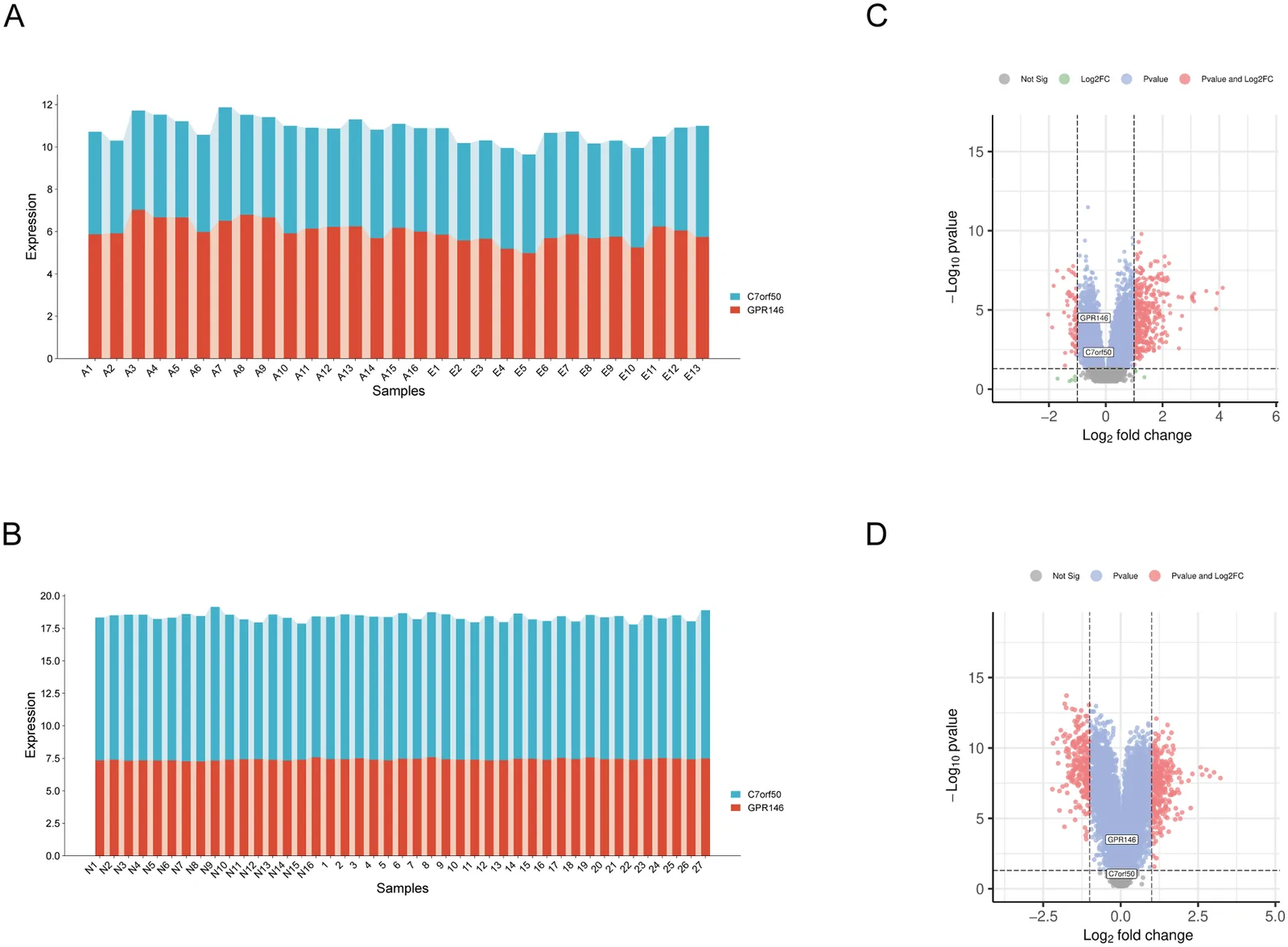
Expression patterns of GPR146 and Cholesin (C7orf50) across atherosclerotic plaque progression and instability. **(A)** Bar graphs showing GPR146 and Cholesin mRNA expression levels in individual samples from the GSE28829 dataset, including early lesions (*n* = 13) and advanced lesions (*n* = 16). **(B)** Bar graphs showing GPR146 and Cholesin mRNA expression levels in individual samples from the GSE163154 dataset, including non-IPH stable plaques (*n* = 16) and IPH unstable plaques (*n* = 27). **(C)** Comparison of GPR146 expression between advanced and early lesions in the GSE28829 dataset. **(D)** Comparison of GPR146 expression between unstable and stable plaques in the GSE163154 dataset. Differentially expressed genes were defined using the following cutoff criteria: |log2FC| > 1 and adjusted *P* *<* 0.05 after Benjamini-Hochberg correction.

We next analyzed the GSE28829 and GSE163154 datasets to assess the expression patterns of Cholesin and GPR146 during plaque progression and instability. GSE28829 was used to compare advanced vs. early atherosclerotic lesions, whereas GSE163154 was used to compare plaques with intraplaque hemorrhage (IPH; unstable plaques) vs. non-IPH plaques (stable plaques). Figures 3A,B show the expression levels of GPR146 and Cholesin in individual samples from these two datasets. Cholesin expression did not differ significantly between advanced and early lesions in GSE28829 or between IPH and non-IPH plaques in GSE163154. In contrast, GPR146 expression was significantly higher in advanced than in early lesions in GSE28829 (*P* < 0.05, Figure 3C) and higher in IPH plaques than in non-IPH plaques in GSE163154 (*P* < 0.05, Figure 3D).

### Functional enrichment analysis of differentially expressed genes reveals the specific pathological processes at different stages of plaques

**Figure 4:**
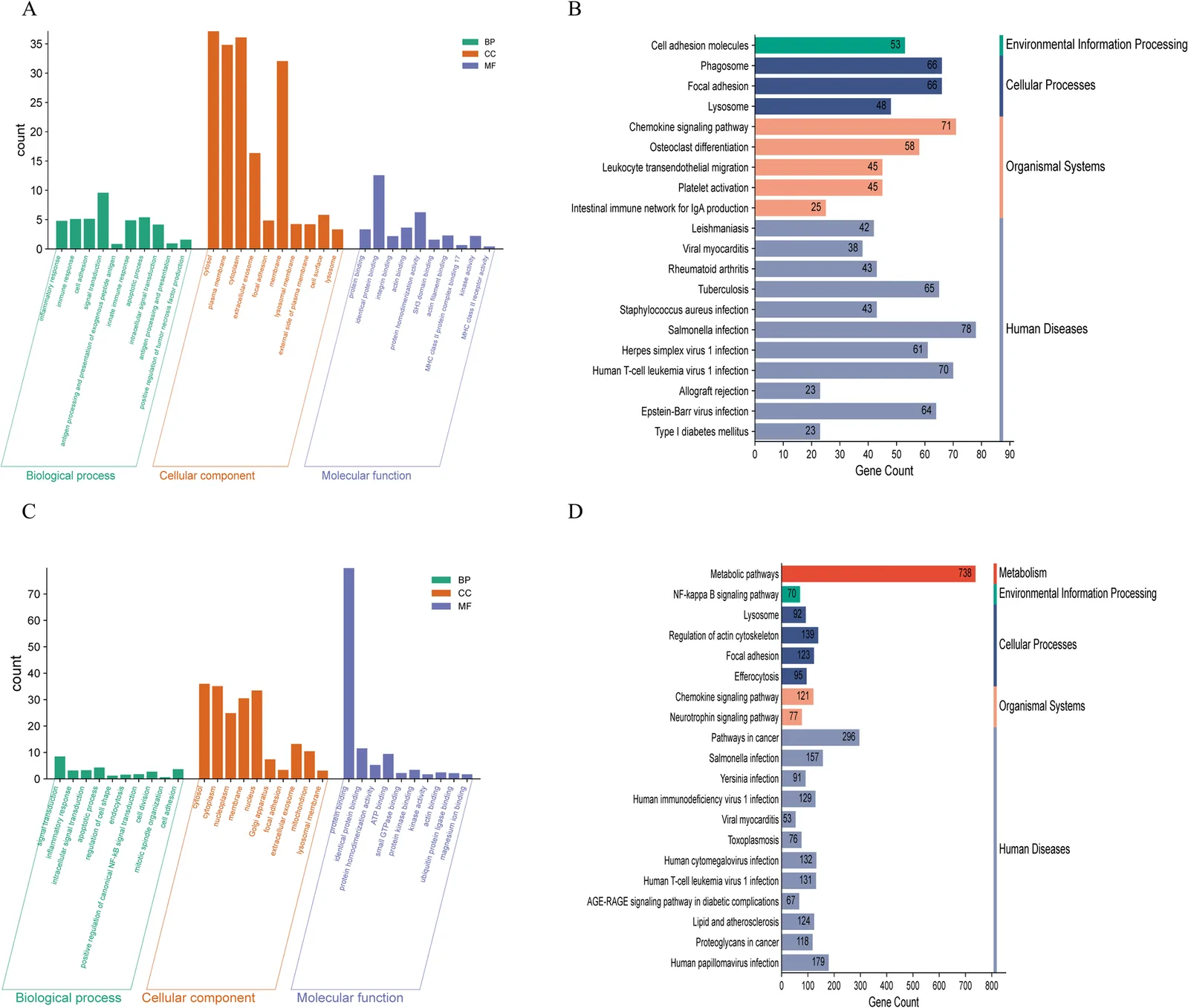
Functional enrichment analysis of differentially expressed genes in the GSE28829 and GSE163154 datasets. **(A)** Top enriched Gene Ontology (GO) terms of differentially expressed genes between advanced and early atherosclerotic lesions in GSE28829. **(B)** Top enriched Kyoto Encyclopedia of Genes and Genomes (KEGG) pathways of differentially expressed genes in GSE28829. **(C)** Top enriched GO terms of differentially expressed genes between intraplaque hemorrhage (IPH) and non-IPH plaques in GSE163154. **(D)** Top enriched KEGG pathways of differentially expressed genes in GSE163154. Enriched terms with *P* *<* 0.05 were considered statistically significant. GO, Gene Ontology; KEGG, Kyoto Encyclopedia of Genes and Genomes.

To explore the biological functions that undergo significant changes at different stages of the disease (progression and instability), we conducted GO and KEGG enrichment analyses on differentially expressed genes in the GSE28829 and GSE163154 datasets. For GO enrichment, we selected the top 10 terms with the highest gene ratio in Biological Process (BP), Cellular Component (CC), and Molecular Function (MF) for each dataset and presented them as bar charts (Figures 4A,C). For KEGG enrichment analysis, we selected the top 20 entries and plotted the bar chart (Figures 4B,D).

As shown in the figure, in GSE28829, the BP category was significantly enriched in cell adhesion, signal transduction, and apoptotic processes. The CC category involved plasma membrane, focal adhesion and cell surface. The MF class was significantly enriched in protein binding, kinase activity and MHC class II receptor activity. KEGG enrichment analysis included pathways such as Chemokine signaling pathway, Leukocyte transendothelial migration, and Cell adhesion molecules.

In addition, in GSE163154, The BP category was significantly enriched in intracellular signal transduction, inflammatory response, and positive regulation of canonical NF-κB signal Related to transduction. The CC category involved membrane, focal adhesion, and extracellular exosome. The MF class was significantly enriched in protein kinase binding and kinase activity. KEGG enrichment analysis included pathways such as Chemokine signaling pathway, NF-κB signaling pathway, and Lipid and atherosclerosis.

### Bioinformatic prediction of GPR146 co-associated functional networks

**Figure 5:**
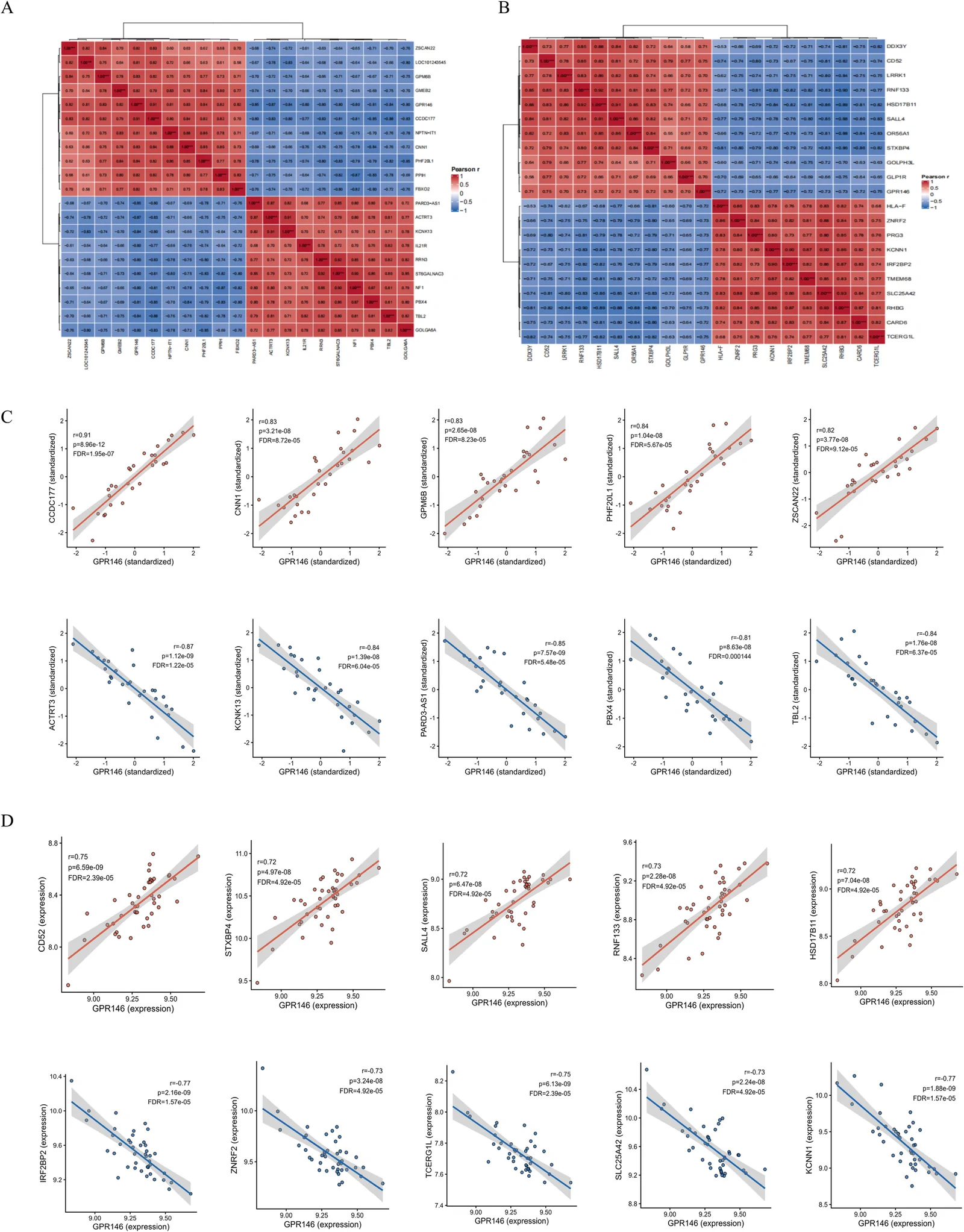
Correlation matrix of GPR146 and top co-expressed genes. **(A)** The top 10 positively and negatively correlated co-expressed genes with GPR146 in the GSE28829 dataset. **(B)** The top 10 positively and negatively correlated co-expressed genes with GPR146 in the GSE163154 dataset. **(C)** The top 5 positively/negatively correlated genes co-expressed with GPR146 in the GSE28829 dataset. **(D)** The top 5 positively/negatively correlated genes co-expressed with GPR146 in the GSE163154 dataset. All reported *P* values from correlation tests were FDR-adjusted using Benjamini-Hochberg method; see Supplementary Table S1 for full *r*, raw *p*, and FDR values.

To explore the transcriptional landscape associated with GPR146, we constructed a correlation matrix using the GSE28829 dataset (Figure 5A) and GSE163154 dataset (Figure 5B). In GSE28829, GPR146 exhibited strong positive correlations with *ZSCAN22* (*r* = 0.82), *LOC101243545* (*r* = 0.81), GMEB2 (*r* = 0.82), CCDC177 (*r* = 0.91), and *GPM6B* (*r* = 0.83). Conversely, it was negatively correlated with PARD3-AS1 (*r* = −0.85), ACTRT3 (*r* = −0.87), KCNK13 (*r* = −0.84), IL21R (*r* = −0.80) and RRN3 (*r* = −0.80). In GSE163154, GPR146 was strongly positively correlated with CD52 (*r* = 0.75), RNF133 (*r* = 0.73), STXBP4 (*r* = 0.72), SALL4 (*r* = 0.72), and HSD17B11 (*r* = 0.72). In contrast, GPR146 was negatively correlated with KCNN1 (*r* = −0.77), IRF2BP2 (*r* = −0.77), TCERG1L (*r* = −0.75), SLC25A42 (*r* = −0.73), and ZNRF2 (*r* = −0.73).

To explore the potential mechanism of GPR146, we constructed its co-expression network and conducted a functional enrichment analysis. In the GSE28829 dataset, genes co-expressed with GPR146 were significantly enriched in entries such as “regulation of transcription by RNA polymerase II” (GO:0006357, *P* = 0.018) and “fibroblast proliferation” (GO:0048144, *P* = 0.014, Table 3). In the GSE163154 dataset, GPR146 co-expressed genes showed non-significant trends toward Wnt- and calcium-related terms, including the “positive regulation of canonical Wnt signaling pathway” (GO:0090263, *P* = 0.059), the “positive regulation of cytosolic calcium ion concentration” (GO:0007204, *P* = 0.071, Table 3). These correlations suggest that GPR146 may be associated with distinct transcriptional contexts across plaque stages, although the causality and regulatory direction remain to be experimentally determined.

**Table 3:** Selected GO terms associated with GPR146 co-expressed genes in GSE28829 and GSE163154.

Ontology	ID	Description	Count	*P*-value
GSE28829				
BP	GO:0006357	Regulation of transcription by RNA polymerase II	40	0.018
BP	GO:0048144	Fibroblast proliferation	22	0.014
BP	GO:0007420	Brain development	22	0.072
MF	GO:0005515	Protein binding	100	0.065
GSE163154				
BP	GO:0090263	Positive regulation of canonical Wnt signaling pathway	18	0.059
BP	GO:0007204	Positive regulation of cytosolic calcium ion concentration	20	0.071

Terms with *P* < 0.05 were considered statistically significant. Terms with *P* ≥ 0.05 are shown as exploratory, non-significant trends and should not be interpreted as significant enrichment.

### GPR146 is upregulated in oxLDL-treated THP-1 monocytes

**Figure 6:**
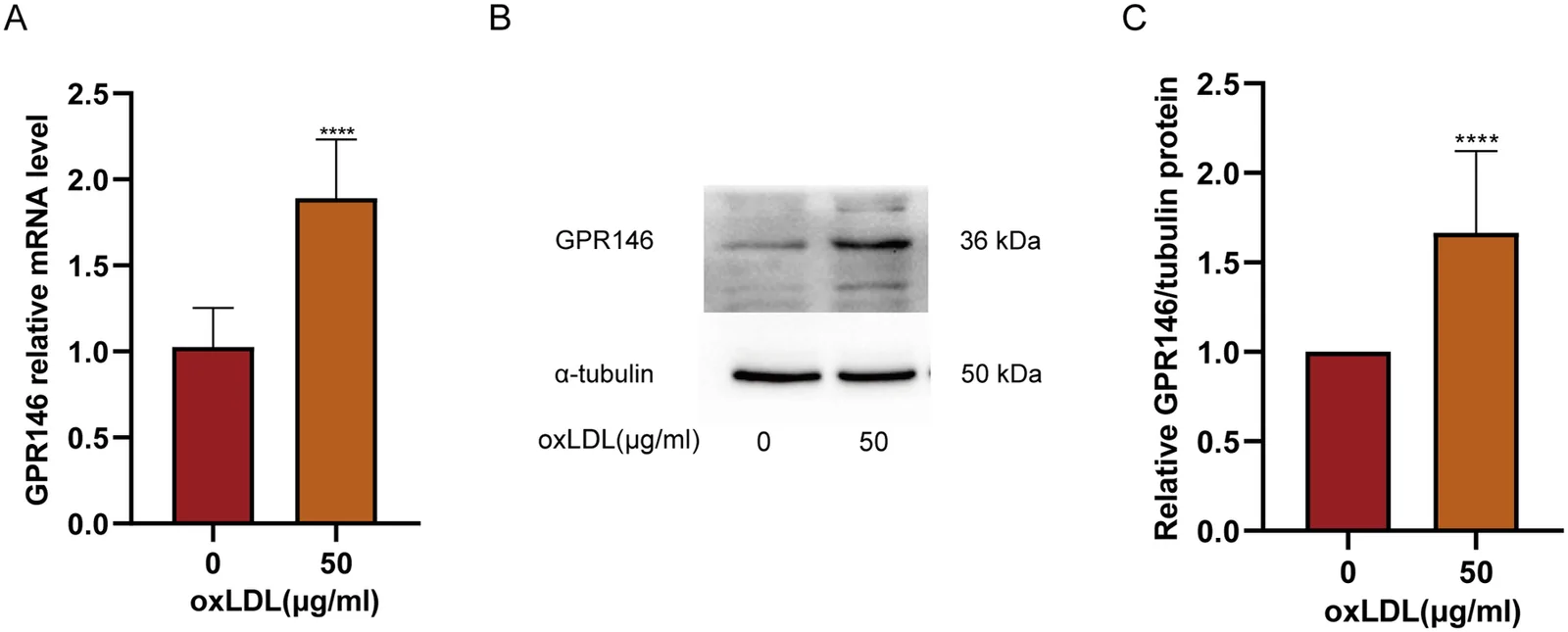
GPR146 is upregulated in oxLDL-treated THP-1 cells. **(A)** RT-qPCR analysis of GPR146 mRNA expression after 50 μg/mL oxLDL for 24 h (*n* = 3); **(B)** Representative western blot and **(C)** quantification of GPR146 protein expression after stimulation with 50 μg/mL oxLDL for 24 h (*n* = 3 independent experiments). Data are shown as mean ± SD. *P *<* 0.05, ****P *<* 0.0001 vs. control (0 μg/mL) by unpaired Student's *t* test.

To investigate whether GPR146 is expressed on monocytes and whether its expression is affected by oxLDL, GPR146 expression levels were measured in THP-1 monocytes at baseline and after oxLDL stimulation. RT-qPCR and Western blot analyses showed that oxLDL stimulation significantly increased GPR146 mRNA and protein expression, respectively (Figures 6A–C).

### Knockdown of GPR146 attenuates oxLDL-induced inflammation

**Figure 7:**
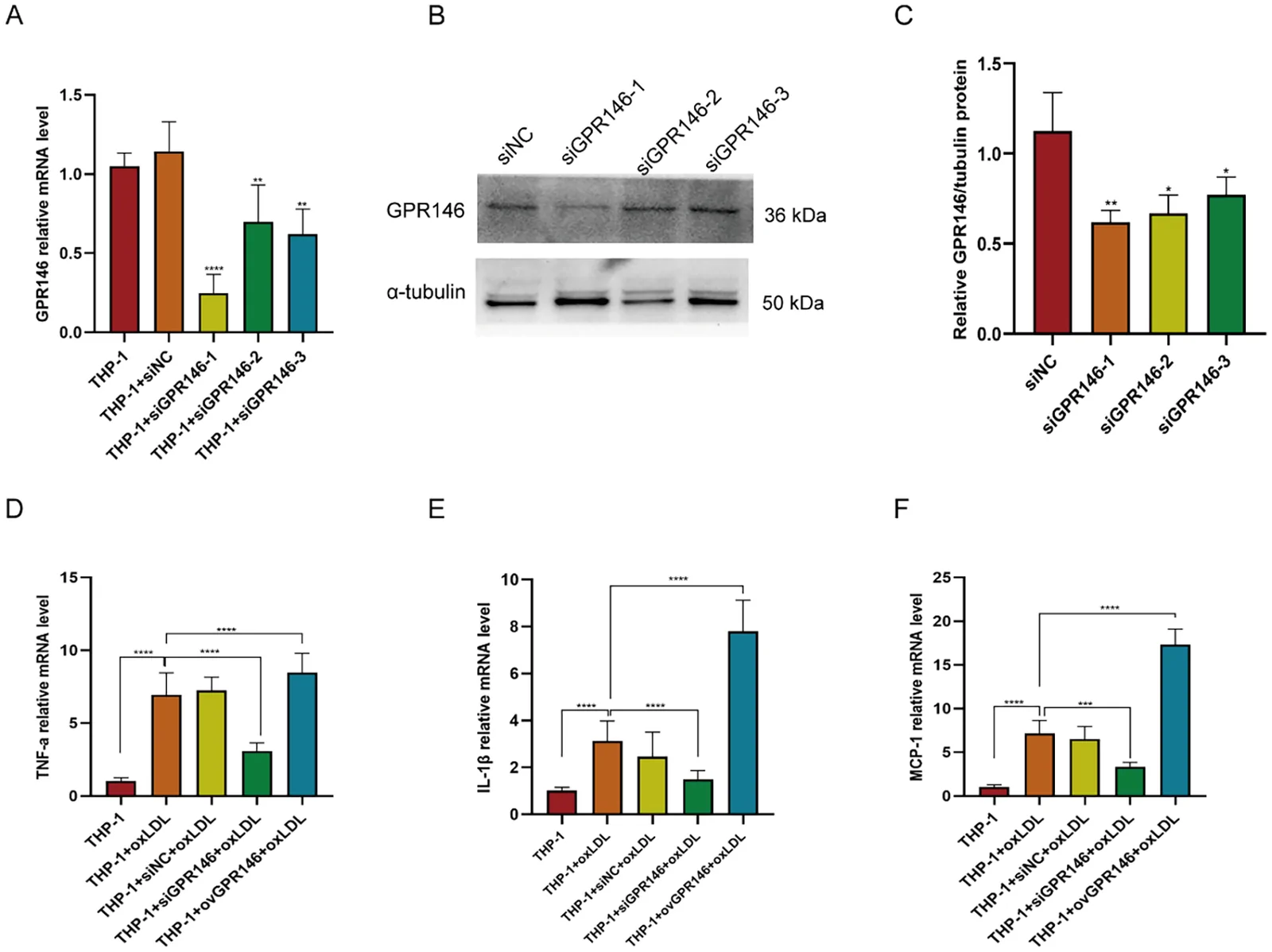
GPR146 knockdown attenuates oxLDL-induced inflammatory gene expression in THP-1 cells. THP-1 cells were transfected with siNC or GPR146 siRNAs (siGPR146-1, siGPR146-2 and siGPR146-3). GPR146 mRNA **(A)** and protein (B and C) levels were analyzed by RT-qPCR and Western blotting, respectively. siGPR146-1, which showed the greatest knockdown efficiency, was used for subsequent functional experiments. For overexpression experiments, cells were transfected with pcDNA3.1-GPR146 or empty vector. After transfection, cells were stimulated with 50 μg/mL oxLDL for 24 h. The mRNA expression levels of TNF-α **(D)**, IL-1β **(E)** and MCP-1 **(F)** were measured by RT-qPCR. Data are presented as mean ± SD from three independent experiments. *P *<* 0.05, **P *<* 0.01, ****P *<* 0.0001 by one-way ANOVA followed by Tukey's *post hoc* test.

To investigate the role of GPR146 in atherosclerosis, THP-1 cells were transfected with three independent GPR146-targeting small interfering RNAs (siRNAs). The results showed that all three siRNAs reduced GPR146 mRNA and protein expression, with siGPR146-1 exhibiting the greatest knockdown efficiency (Figures 7A–C). Therefore, siGPR146-1 was selected for subsequent functional experiments. Total RNA was extracted from THP-1 cells 24 h after the indicated treatments. oxLDL treatment significantly increased the expression of TNF-α, IL-1β, and MCP-1 in the cells (Figures 7D–F). Compared with siNC, GPR146 silencing significantly attenuated the oxLDL-induced expression of these inflammatory genes (*P* < 0.05). Conversely, GPR146 overexpression significantly increased the mRNA expression of TNF-α, IL-1β, and MCP-1. These findings support a role for GPR146 in modulating oxLDL-induced inflammatory gene expression in THP-1 cells.

## Discussion

By integrating clinical samples, public transcriptomic datasets, and *in vitro* experiments, this study investigated the potential relevance of Cholesin and its receptor GPR146 to atherosclerotic plaque phenotypes. The main findings are as follows: (1) PBMC Cholesin mRNA was significantly lower in the unstable-plaque group than in the stable-plaque and control groups (stable ≈ control > unstable). (2) GPR146 expression was increased in advanced and unstable plaques; (3) GPR146 co-expression analyses identified transcription-related associations in advanced plaques and exploratory Wnt- and calcium-related trends in IPH-associated plaques; (4) GPR146 knockdown attenuated oxLDL-induced inflammatory gene expression in THP-1 cells. Collectively, these findings suggest that PBMC Cholesin and plaque GPR146 may be associated with distinct aspects of plaque phenotypes, although their functional relationship in local plaque biology remains to be determined.

The PBMC and plaque-tissue findings showed distinct association patterns across different biological compartments and non-paired cohorts. PBMC Cholesin mRNA was lower in participants with unstable plaques than in those with stable plaques and angiographic controls without significant coronary stenosis. In contrast, plaque-tissue Cholesin expression was reduced in atherosclerotic lesions compared with control vessels but did not differ between early and advanced lesions or between IPH and non-IPH plaques. Whether this apparent discordance reflects compartment-specific regulation, differences in cellular composition, cohort characteristics, or methodological heterogeneity cannot be determined from the present data. Because plasma Cholesin protein was not measured and the PBMC and tissue samples were not paired, these findings should not be interpreted as evidence of a unified systemic–local regulatory mechanism. Rather, PBMC Cholesin mRNA may be associated with systemic metabolic or inflammatory status, whereas the biological significance of reduced plaque-tissue Cholesin remains uncertain.

Previous studies provide a biological context for these observations. Hu et al. demonstrated that Cholesin binds to hepatic GPR146 and suppresses the PKA-ERK1/2-SREBP2 pathway, thereby reducing cholesterol synthesis. They further showed that exogenous Cholesin supplementation also reduced atherosclerotic plaque burden in high-fat-fed LDLR-deficient mice (7). These findings support a model in which Cholesin is secreted in response to elevated cholesterol levels and exerts a protective metabolic effect through inhibition of GPR146 signaling. In plaque tissue, however, Cholesin expression was lower in atherosclerotic lesions than in control vessels in GSE100927, but did not differ significantly between early and advanced lesions in GSE28829 or between stable and unstable plaques in GSE163154. Thus, although plaque-tissue Cholesin appears to be reduced in established atherosclerotic lesions, the available data do not support a clear association with subsequent lesion progression or plaque vulnerability. Accordingly, plaque-tissue Cholesin did not distinguish lesion stage or stability in the datasets analyzed. Taken together, the divergent PBMC and plaque-tissue expression patterns suggest that Cholesin expression these two compartments may be regulated through distinct mechanisms and may reflect parallel rather than convergent biological processes.

PBMC Cholesin mRNA was higher in the stable-plaque and control groups than in the unstable-plaque group, suggesting an association between lower PBMC Cholesin expression and plaque instability in this cohort. However, whether this association is independent of clinical covariates or predictive of future cardiovascular events remains unknown. One possible explanation is that PBMC Cholesin expression reflects systemic metabolic or inflammatory status rather than local plaque pathology, although its relationship with circulating Cholesin protein requires further validation. Previous studies have shown that Cholesin suppresses hepatic GPR146-PKA-ERK1/2-SREBP2 signaling and lowers cholesterol levels (7). Consistent with this pathway, GPR146 deficiency reduces plasma cholesterol in both wild-type and LDLR-deficient mice (12), while rare GPR146 variants in humans are associated with lower cholesterol levels (13). Nevertheless, whether PBMC Cholesin expression reflects this systemic pathway remains uncertain. In the present study, GPR146 expression was higher in advanced than in early plaques and in IPH than in non-IPH plaques, supporting an association with advanced or vulnerable plaque phenotypes. However, the current data cannot determine whether GPR146 contributes causally to plaque destabilization or merely reflects disease severity.

**Figure 8:**
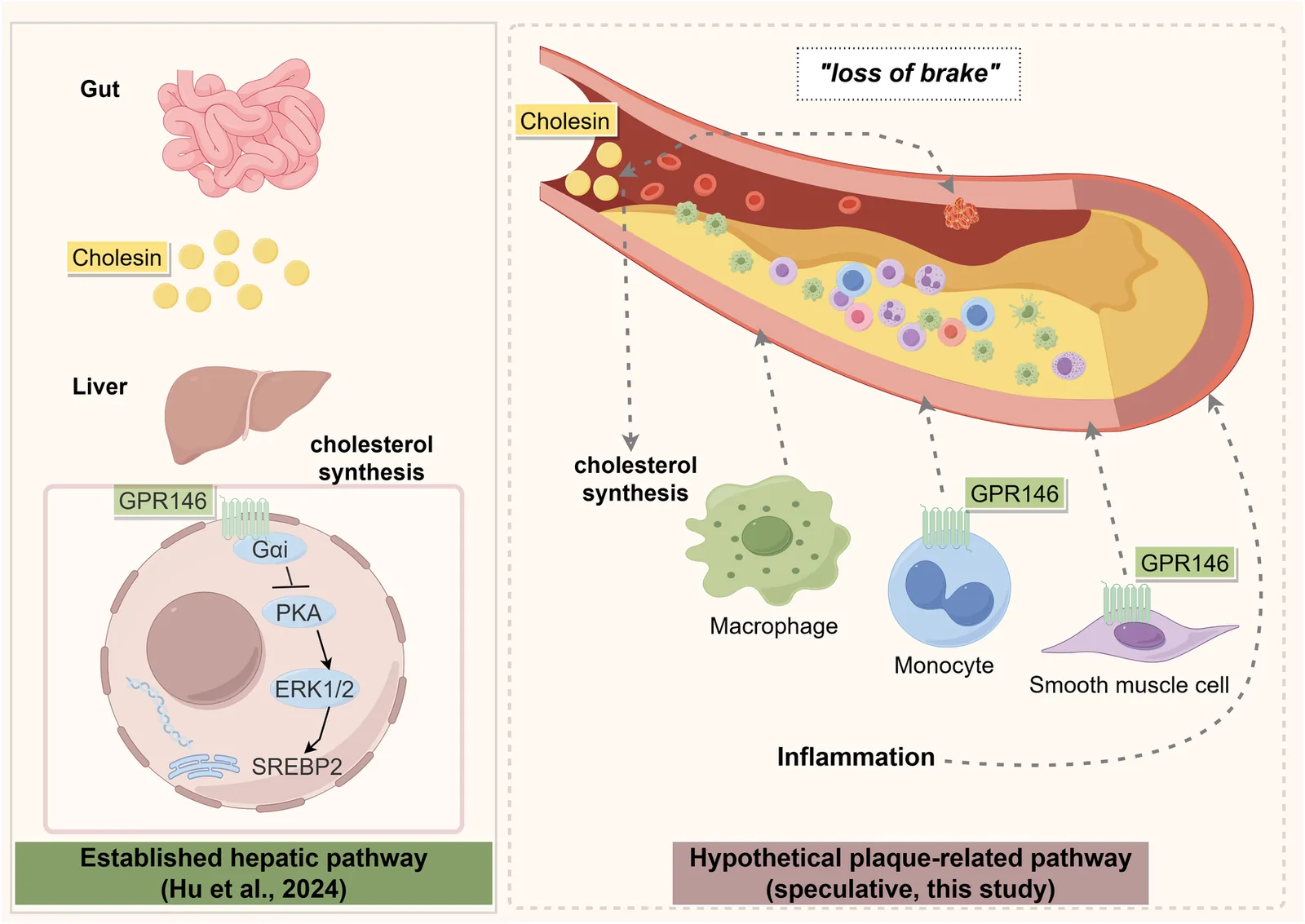
Proposed Cholesin–GPR146 “loss-of-brake” model in atherosclerosis. The left panel shows the established hepatic Cholesin–GPR146 pathway. Cholesin secreted by the small intestine binds to hepatic GPR146, inhibits downstream PKA-ERK1/2-SREBP2-mediated signaling, and suppresses hepatic cholesterol synthesis (7). The right panel illustrates a hypothesis-generating plaque-related model involving GPR146-expressing macrophages, vascular smooth muscle cells, and circulating monocytes. In this model, lower PBMC Cholesin mRNA and higher plaque GPR146 expression may reflect reduced Cholesin-mediated restraint and enhanced GPR146-associated inflammatory activity during plaque progression and destabilization. Solid lines indicate established relationships, whereas dashed lines indicate hypothetical relationships requiring further validation. The figure was created using Figdraw.

Direct evidence for Cholesin-GPR146 interaction in plaque tissue is currently lacking. Hu et al. demonstrated that gut-derived Cholesin selectively binds to GPR146 on hepatocytes, but whether this interaction occurs in plaque-resident macrophages, smooth muscle cells, or endothelial cells remains unknown. Based on the lower PBMC Cholesin mRNA observed in participants with unstable plaques, the higher GPR146 expression detected in advanced and IPH-associated plaques, and the pro-inflammatory effects associated with GPR146 in THP-1 monocytes, we propose a hypothesis-generating “loss-of-brake” model (Figure 8). In this model, reduced Cholesin-related inhibitory signaling may coexist with enhanced GPR146-associated inflammatory activity during plaque progression and destabilization. However, PBMC Cholesin mRNA has not been shown to reflect circulating Cholesin protein, and direct Cholesin binding to or functional inhibition of GPR146 in plaque-resident cells was not examined. Therefore, this model should not be interpreted as an established systemic–local mechanism but rather as a conceptual framework for future cell-type-specific and *in vivo* validation.

The co-expression analysis of GPR146-associated genes provided exploratory insights into the transcriptional context of plaque stages and instability. Among the GPR146 co-expressed genes, IRF2BP2 was notable for its strong negative correlation with GPR146 in GSE163154 (*r* = −0.77). Previous studies have shown that macrophage-specific loss of IRF2BP2 exacerbates atherosclerosis, suggesting a potential association between GPR146-related transcriptional patterns and macrophage inflammatory phenotypes (7, 14). However, this correlation does not establish a direct regulatory relationship between GPR146 and IRF2BP2. Similarly, although KCNN1 was negatively correlated with GPR146, this transcriptional association should not be interpreted as evidence that GPR146 functionally suppresses KCNN1 or directly regulates vascular electrophysiology. GO enrichment reflects the collective functional profile of the co-expression gene set rather than the regulatory direction of any individual gene, and transcriptomic data alone cannot determine the net effect on intracellular calcium signaling. GPM6B, another co-expressed gene, has been associated with vascular morphogenesis and smooth muscle cell and endothelial cell proliferation (15), although the biological significance of its association with GPR146 remains unknown. These findings therefore provide testable hypotheses rather than evidence of direct gene–gene regulation.

Functional enrichment analysis provided additional exploratory information regarding the transcriptional context of GPR146. In GSE28829, GPR146-associated genes were significantly enriched for terms including “regulation of transcription by RNA polymerase II” and “fibroblast proliferation,” suggesting a possible association with transcriptional remodeling in advanced lesions. In GSE163154, however, Wnt- and calcium-related terms showed only non-significant trends and should therefore be interpreted cautiously. Independently, our THP-1 experiments showed that GPR146 silencing attenuated oxLDL-induced expression of TNF-α, IL-1β, and MCP-1, supporting a role for GPR146 in modulating inflammatory gene expression in monocytes. These experiments did not validate the Wnt- or calcium-related associations identified in the transcriptomic analyses. Although Wnt/β-catenin and calcium-dependent Wnt signaling have been implicated in endothelial dysfunction, macrophage activation, and smooth muscle cell phenotypic remodeling during atherosclerosis (16–19), the present data do not establish a functional link between GPR146 and these pathways. Accordingly, these enrichment results should be regarded as hypothesis-generating and require direct validation in relevant plaque-resident cell types.

Atherosclerosis is mainly characterized by lipid deposition in large and medium arteries, which gradually forms lipid streaks, fibrous plaques, atherosclerotic plaques, and even complex lesions. Its formation process mainly involves endothelial dysfunction, monocyte chemotaxis, macrophage polarization and smooth muscle cell migration (20–23) Previous studies have found that GPR146 is expressed not only on liver cell membranes but also in smooth muscle cells, macrophages and endothelial cells (7, 24–26) While this broad expression pattern raises the possibility that GPR146 could contribute to plaque instability through multiple cellular mechanisms, our functional data are limited to THP-1 monocytes, and we cannot extrapolate these *in vitro* findings to plaque-resident smooth muscle cells or endothelial cells where GPR146 is also expressed without lineage-specific validation.

We used THP-1 monocytes as an *in vitro* model to examine GPR146 expression under basal conditions and after oxLDL stimulation. oxLDL is a major atherogenic stimulus that promotes monocyte/macrophage activation and contributes to development and progression of atherosclerosis (27). In our study, oxLDL significantly increased GPR146 expression, together with the mRNA expression of TNF-α, IL-1β, and MCP-1. TNF-α and IL-1β are key pro-inflammatory cytokines involved in macrophage activation and the amplification of vascular inflammation (28, 29), whereas MCP-1 promotes monocyte recruitment, adhesion, and foam-cell formation, thereby contributing to plaque progression (30). These findings support a role for GPR146 in modulating pro-inflammatory cytokine mRNA expression in monocytes and warrant further investigation of GPR146 as a candidate regulator of monocyte-lineage inflammatory responses.

The potential mechanisms through which GPR146 modulates inflammatory responses in monocytes warrant further consideration. The cAMP/PKA signaling pathway is a well-recognized negative regulator of NF-κB-mediated inflammation in macrophages (31). Elevation of intracellular cAMP activates PKA, which in turn phosphorylates CREB; activated CREB competes with NF-κB for limiting amounts of transcriptional co-activators such as CBP/p300, thereby suppressing the transcription of pro-inflammatory cytokines including TNF-α, IL-1β, and MCP-1 (32). Conversely, reduced cAMP/PKA signaling relieves this brake, enhancing inflammatory gene expression. The G-protein coupling of GPR146 in monocytes remains unclear, including whether it preferentially signals through Gαi or Gαs. In our study, GPR146 silencing attenuated oxLDL-induced inflammatory gene expression, raising the possibility that GPR146 may influence the cAMP/PKA pathway (33, 34). However, this interpretation remains indirect because cAMP levels, PKA activity, and NF-κB signaling were not measured. Other pathways, including NLRP3/caspase-1 inflammasome signaling and ERK-dependent mechanisms, may also contribute to the observed effects (12, 34).

Beyond canonical signaling, the potential role of GPR146 should also be considered within the broader context of macrophage immunometabolism in atherosclerosis (35). Macrophages in atherosclerotic plaques undergo extensive metabolic reprogramming in response to atherogenic stimuli such as modified lipoproteins and hypoxia, involving alterations in glycolysis, oxidative phosphorylation, lipid metabolism, and amino acid metabolism (36, 37). These metabolic changes influence macrophage activation and polarization and thereby contribute to atherosclerotic progression (38). Lipid metabolism is particularly relevant, as enhanced fatty acid synthesis is associated with pro-inflammatory macrophage phenotypes and altered cholesterol homeostasis (39). Although these pathways were not directly examined in the present study, the attenuation of oxLDL-induced inflammatory gene expression after GPR146 silencing raises the possibility that GPR146 may intersect with macrophage metabolic reprogramming. Given the established link between hepatic GPR146 signaling and SREBP2 activity, GPR146 may influence monocyte inflammatory responses through lipid-metabolic pathways; however, this hypothesis requires direct experimental validation.

In addition to metabolic reprogramming, efficient macrophage efferocytosis, which mediates the clearance of apoptotic cells, is another important determinant of plaque stability (40, 41). Impaired efferocytosis promotes apoptotic cell accumulation, secondary necrosis, and necrotic core expansion, thereby contributing to plaque destabilization. Recent studies have highlighted epigenetic regulation of efferocytosis, with RBPJ, a Notch signaling mediator, promoting apoptotic cell clearance through epigenetic modifications (42). Whether GPR146 signaling interacts with these efferocytic pathways remains unknown and warrants future investigation.

Overall, lower PBMC Cholesin mRNA was associated with the unstable-plaque phenotype, whereas plaque GPR146 expression was higher in advanced and IPH-associated lesions. GPR146 silencing also attenuated oxLDL-induced inflammatory gene expression in THP-1 monocytes. However, the present observational and *in vitro* findings do not establish a direct Cholesin–GPR146 interaction in plaque tissue or demonstrate that GPR146 causally drives plaque instability. Further validation using paired clinical samples, lineage-specific cellular models, and *in vivo* approaches is required.

## Conclusion

In conclusion, our integrated clinical, bioinformatic, and *in vitro* analyses suggest distinct expression patterns and associations of Cholesin and GPR146 in atherosclerosis. PBMC Cholesin expression was associated with plaque stability, whereas local plaque Cholesin expression did not distinguish stable from unstable lesions. In contrast, GPR146 was upregulated in advanced and vulnerable plaques, and its silencing attenuated oxLDL-induced inflammatory gene expression in THP-1 monocytes. These findings support GPR146 as a candidate molecule associated with plaque progression and instability. However, its causal role in plaque destabilization and its therapeutic relevance require further validation in experimental models.

### Limitations

Several limitations of this study should be acknowledged. First, and most importantly, direct evidence for Cholesin-GPR146 interaction in atherosclerotic plaque tissue is currently lacking. Whether Cholesin binds to or functionally modulates GPR146 in plaque-resident vascular cells, including macrophages, smooth muscle cells, or endothelial cells, remains unknown. Therefore, the proposed “loss-of-brake” model remains speculative and should be considered hypothesis-generating rather than conclusive.

Second, although this study integrates clinical samples, public transcriptomic datasets, and *in vitro* experiments, the mechanistic role of GPR146 in plaque progression and instability remains insufficiently validated *in vivo*. The co-expression networks identified in this study should be interpreted as predictive associations rather than direct regulatory interactions. Future studies using animal models and cell-type-specific approaches are required to clarify whether GPR146 actively contributes to plaque destabilization or merely reflects disease severity.

Third, Cholesin expression was measured at the mRNA level in PBMCs rather than at the protein level in plasma. While we observed a significant association between PBMC Cholesin mRNA and plaque stability, the relationship between PBMC Cholesin mRNA and circulating Cholesin protein remains unknown. Future studies measuring plasma Cholesin protein using a validated ELISA are needed to validate our findings and determine whether PBMC Cholesin mRNA reflects systemic Cholesin status. Additionally, the compartment-specific interpretation of PBMC and tissue Cholesin expression is derived from our clinical PBMC cohort and publicly available tissue transcriptomic datasets, which were not matched at the individual level. Validation in paired PBMC and plaque specimens from the same patients is warranted in future studies.

Fourth, the association between PBMC Cholesin expression and plaque stability was based on a cross-sectional clinical cohort and should be interpreted with caution. Larger prospective studies are required to evaluate whether PBMC Cholesin expression has independent predictive value for plaque instability or future cardiovascular events.

Fifth, although three independent siRNAs were screened for GPR146 knockdown efficiency, the downstream inflammatory experiments were performed only with siGPR146-1. Therefore, sequence-specific off-target effects cannot be completely excluded. Validation using a second siRNA and rescue experiments with an siRNA-resistant GPR146 construct would further strengthen the specificity of the observed phenotype.

### Future directions

While these findings suggest a potential association between the Cholesin-GPR146 axis and plaque phenotype, future studies are needed to functionally validate the proposed mechanisms. In particular, animal models of atherosclerosis and cell-type-specific approaches will be important to determine whether GPR146 actively contributes to plaque progression or instability, or merely reflects disease severity. Future work should also assess whether Cholesin directly modulates GPR146-mediated inflammatory signaling in plaque-resident vascular cells. In addition, measuring circulating Cholesin protein levels in larger prospective cohorts will help determine whether PBMC Cholesin expression has independent predictive value for plaque instability or future cardiovascular events.

## Data Availability

The original contributions presented in the study are included in the article/Supplementary Material, further inquiries can be directed to the corresponding author.
